# The monoaminergic systems as drivers of Alzheimer's disease pathophysiology and symptomatology

**DOI:** 10.1097/WCO.0000000000001455

**Published:** 2026-01-28

**Authors:** Heidi I.L. Jacobs

**Affiliations:** a Athinoula A. Martinos Center for Biomedical Imaging, Department of Radiology, Massachusetts General Hospital and Harvard Medical School, Boston, Massachusetts, USA; b Faculty of Health, Medicine and Life Sciences, Mental Health and Neuroscience Research Institute, Alzheimer Centre Limburg, Maastricht University, Maastricht, The Netherlands

**Keywords:** locus coeruleus, norepinephrine, raphe nucleus, serotonin, tau

## Abstract

**Purpose of review:**

To summarize recent animal, postmortem and in vivo human studies examining the role of the noradrenergic and serotonergic system in the pathophysiology and symptomatology of Alzheimer's disease (AD).

**Recent findings:**

Early in adulthood, the locus coeruleus and raphe nucleus accumulate tau, undergo morphological changes, and exhibit hyperexcitability, which contributes to the development of neuropsychiatric symptoms. As cortical AD pathology increases, these nuclei become hypoactive, but elevated neurotransmitter levels persist in the cortex, presumably driving amyloid-related hyperexcitability and contributing to tau spreading and cognitive decline.

**Summary:**

The pathologic changes occurring within these monoaminergic systems temporally align with the observation that neuropsychiatric symptoms precede cognitive changes in AD, indicating that these systems link the earliest pathobiology of the disease to the evolution of the symptoms. The proposed monoaminergic framework intends to guide researchers into investigating the temporal dynamics between monoaminergic changes, AD pathology, and symptoms, with the ultimate goal of evaluating and developing effective precision therapeutic approaches taking into account the disease stage and symptom profile.

## INTRODUCTION

Alzheimer's disease (AD) is neuropathologically characterized by beta-amyloid (Aβ) plaques and hyperphosphorylated tau forming pretangle material and neurofibrillary tangles upon aggregation [1,2]. These plaques and tangles progress in a predictable topographical pattern in the cortex, as described in the seminal Thal and Braak stages, respectively [3]. However, autopsy studies reported that the neuromodulatory subcortical system (NSS) nuclei in the brainstem and basal forebrain are among the first regions to accumulate hyperphosphorylated tau, preceding detectable levels of cortical Aβ or tau and the emergence of cognitive symptoms [4–9].

The NSS represents a phylogenetically conserved network of nuclei that release neurotransmitters, neuropeptides, or hormones through widespread projections, allowing them to modulate cognitive processes and behaviors. A growing body of evidence from animal and human neuroimaging studies demonstrates that this early vulnerability to pathology impacts the morphology and function of these nuclei, collectively disrupting the precision with which these systems modulate cortical target regions and circuits, and ultimately drive the evolution of clinical symptoms that define AD [4]. Understanding this clinicopathologic alignment is crucial for identifying predictive biological biomarkers to guide risk triage and therapeutic target selection in primary prevention strategies, a time window where symptom profiles are subtle and heterogeneous. 

**Box 1:**
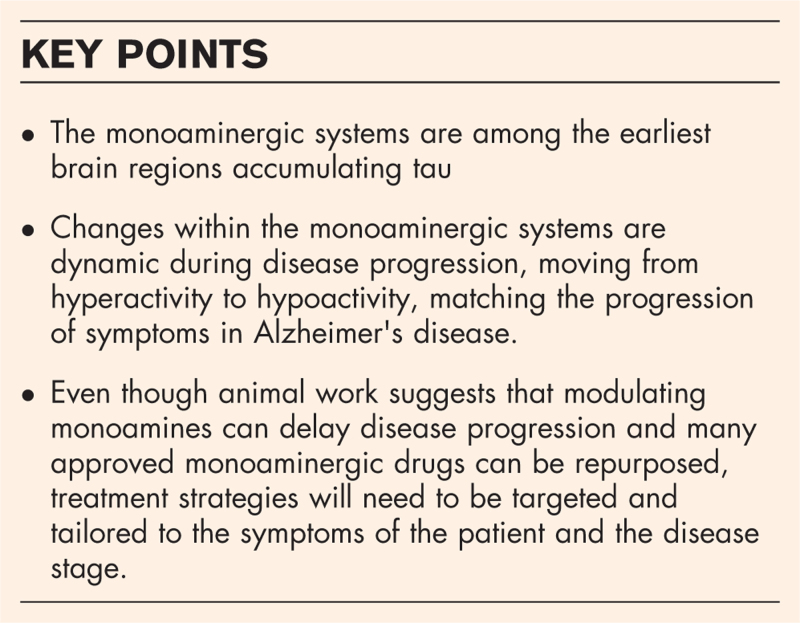
no caption available

**FIGURE 1:**
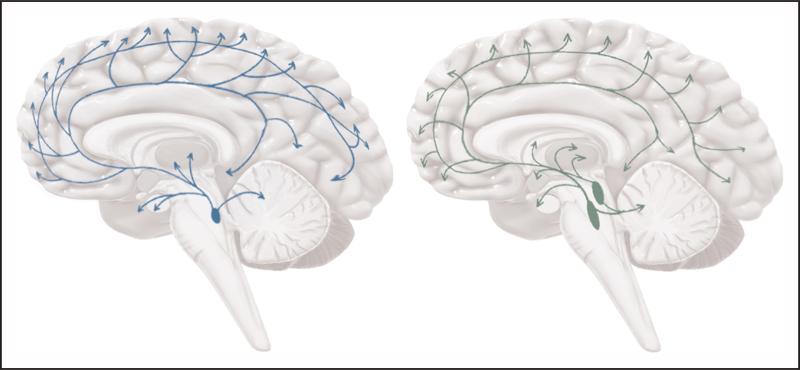
The noradrenergic and serotonergic systems. The noradrenergic (blue) and serotonergic (green) systems send widespread projections to the brain via the locus coeruleus and the dorsal and medial raphe nuclei, respectively.

To illustrate the clinical significance of the NSS throughout the evolution of the disease, we provide a temporal framework describing changes in two key monoaminergic nuclei, the noradrenergic locus coeruleus (NE, LC) and the serotonergic raphe nuclei (5-HT, RN), before, during and after the preclinical or asymptomatic stages of the disease (Fig. 1).

## PRIOR TO THE PRECLINICAL STAGES OF ALZHEIMER'S DISEASE (“PRE-PRECLINICAL”)

Neuropathological studies reported that neurons within the LC and RN are selectively vulnerable to accumulate hyperphosphorylated tau early in the disease process (Braak stages a-c) [4,9,10^▪▪^,^11^,^12^]. By age 50, tau pretangle material is present in these nuclei across all autopsy case [2,5,13,14^▪▪^]. These pretangles, and also their oligomeric forms, may be more toxic than the fully formed tau tangles [15]. Volume of the LC or RN is inversely correlated with Braak stages, starting from Braak stage 0; however, loss of neurons has not been reported prior to Braak stage IV [16]. This suggests that LC and RN neurons undergo various morphological changes in response to tau, including loss of dendritic arborization [17].

In vivo neuroimaging studies have corroborated these autopsy findings, reporting microstructural changes in the LC-entorhinal cortex tract that distinguish individuals in Braak stage 0 from higher stages, as measured with tau-PET [18]. Lower microstructural properties of this tract also correlated with elevated plasma ptau and GFAP markers [19]. While older animal studies linked LC neurodegeneration to neuroinflammation and astrocytic reactivity [20,21], these neuroimaging findings suggest that neuroinflammatory processes begin before neurodegeneration. Indeed, AD mice models with dorsal RN tau at 3 months of age exhibited higher astrocyte density, GFAP reactivity, and neuroinflammation in the dorsal RN neurons [14^▪▪^].

Although the LC and RN avoid cell loss early in the disease process [22] this does not guarantee intact cell function [23]. Phosphorylated tau in the RN or LC of 3-month-old AD model mice was associated with hyperexcitability, increased firing rates, synaptic dysfunction and increases in neurotransmitter release [14^▪▪^,^24^]. This hyperexcitability suggests a shift in the excitatory/inhibitory input balance: glutamatergic transmission was enhanced, which can be excitotoxic [25], and inhibitory GABA receptor expression was reduced (likely from the pericoerulear dendritic region [26]).

Supported by the observations reviewed here, we challenge the notion that these early changes in LC or RN structure and function are merely age-related; instead, these changes dovetail with the progression of pathology and behavior in AD. Animal studies showed that tau in the dorsal RN spreads to regions colocalized with high serotonin reuptake transporter density, including the thalamus, hypothalamus and amygdala [14^▪▪^]. Similarly, tau in LC neurons spreads to the RN and subsequently to medial temporal regions [27,28]. In young mice, RN tau was associated with reduced social interactions and increased depressive-like and anxiety-like behaviors, but no memory deficits [14^▪▪^], paralleling observations that neuropsychiatric symptoms precede cognitive deficits in AD and are linked to the earliest tau depositions in the brain [29].

In vivo human studies echoed these observations. Lower MRI-derived LC integrity predicts subsequent tau spreading to medial temporal lobe areas three years later [30^▪▪^]. Furthermore, among Aβ-negative cognitively normal older individuals, lower LC integrity outperforms standard biomarkers, such as Aβ-PET and hippocampal volume, in predicting elevated entorhinal tau and clinical progression over 6 years [31]. Behaviorally, lower LC integrity was associated with a higher frequency of nocturnal awakenings in asymptomatic older individuals [32]. Additionally, higher social activity exposure was associated with lower postmortem LC degeneration in cognitively normal individuals [33]. In vivo human evidence for the RN is limited, but higher serotonin synthesis in the dorsal RN using [^18^F]fluoro-*m*-tyrosine PET predicted greater cortical atrophy and depressive symptoms in older individuals [34^▪^].

Together, these findings demonstrate that tau-related dysfunction in the LC and RN begins decades before the onset of clinical symptoms, manifesting as early hyperexcitability with morphological changes and initial tau spreading. Critically, these alterations are measurable in vivo and presage disease progression prior to the emergence of amyloidosis, positioning monoaminergic integrity as a promising early risk marker for preclinical AD that may also facilitate triaging individuals for primary prevention strategies.

## THE PRECLINICAL STAGES OF ALZHEIMER'S DISEASE

The preclinical stages of AD, a time when Aβ becomes detectable, and individuals remain asymptomatic, but may experience subtle cognitive changes, coincides with cell loss in the LC and DR nuclei [35], as well as spreading of tau from the medial temporal lobe to neocortical regions [5,16]. While Aβ-related tau spreading has been related to increased neuronal excitability [36], the evolution and impact of LC or RN activity on AD's pathophysiology remains elusive.

Comparing disease stages between animal models and humans is complex. To approach the human preclinical stages, older animals or those with Aβ deposition are considered to reflect preclinical stages, whereas those with high Aβ and/or tau, and cognitive deficits, are included in the next section.

Tau transgenic mice treated with reboxetine, a NE reuptake inhibitor, for two months, showed accelerated tau aggregation in the LC and hippocampus, neuronal and synaptic loss in the hippocampus, and cognitive deficits [37^▪^], suggesting that prolonged exposure to excessively elevated NE is detrimental. Further activity analyses revealed that elevated levels of NE enhanced activation of the PKA and GSK3β kinases [38]. Previous work in AD models showed that NE enhances the binding affinity of oligomeric Aβ to α-2 receptors activating the GSK3β pathway promoting tau hyperphosphorylation [39]. It is conceivable that these high NE levels are associated with an imbalance in neuronal firing modes. LC neurons can fire in brief high-frequency bursts, phasic firing, in response to arousal, novelty or effort, or fire in tonic mode, a low-frequency baseline firing associated with general arousal. An optimal balance between these two modes is pivotal for optimal cognitive performance. Elevated tonic activity of tau-positive LC neurons was associated with anxiety, learning impairments and reductions in NE-fibers in rodents [40].

Changes in NE signaling and the arousal state can result in fragmented sleep, as NE plays a crucial role in sleep-wake transitions, glymphatic clearance of AD pathology and memory consolidation [41]. The importance of sleep-related dysfunctional levels of NE for AD pathology was elegantly demonstrated by showing that mice with chronic sleep disruption exhibited increased formation of Aβ and tau in the LC [42^▪▪^]. While these animal studies highlight the role of (dysfunctional or elevated) NE in tau formation, sleep disturbances, and cognitive decline, work in 15-month-old TgF344AD rats with Aβ observed that somewhere during this phase of the disease, LC neurons switch from a hyperactive to a hypoactive state [43].

While RN neurons exhibited early increased excitability, the behavior of RN neurons during disease progression remains unclear. Examination of the RN in tau models suggested that initial increased excitability of RN neurons is followed by loss of neuronal excitability, loss of 5-HT innervations of cortical regions, and, when neurodegeneration ensues, also increased depression-like behaviors [44]. The finding that escitalopram, a selective serotonin reuptake inhibitor, reduced Aβ-related tau hyperphosphorylation in the hippocampus through specific 5-HT receptors regulating the Akt/GSK3β pathway confirms loss of 5-HT signaling in preclinical AD [45].

Among individuals with preclinical AD, elevated LC FDG-PET signal, reflecting glucose metabolism, was associated with attenuated memory decline [46]. Conversely, lower novelty-related LC BOLD activity (a proxy for phasic activity) was associated with higher entorhinal tau and faster memory decline in preclinical AD [47]. Similarly, lower LC-related catecholaminergic synthesis was associated with higher amygdala tau when cortical network activity was elevated in preclinical AD [48]. Lower LC-related catecholaminergic synthesis was previously also associated with higher neuroticism and depression [49].

The preclinical stage thus represents a critical transition point, where initial hyperactivity gives way to emerging hypoactivity in monoaminergic nuclei, potentially reflecting a window during which secondary prevention interventions might delay disease progression.

## THE SYMPTOMATIC STAGES OF ALZHEIMER'S DISEASE

Clinicopathologic data revealed that the proportion of cases with cognitive impairment increases substantially once Aβ-positive individuals reach Braak stage III/IV and beyond [50]. During this stage, LC neurodegeneration and cell loss become more evident, manifesting as loss of neuromelanin pigmentation at autopsy. Lower pigmentation at autopsy was associated with antemortem greater cognitive impairment, psychiatric symptoms, and more severe sleep fragmentation in patients with mild cognitive impairment (MCI) and AD [51^▪^,^52^,^53^]. In vivo neuroimaging studies similarly found lower MRI-derived LC integrity in AD compared to controls, and in early-onset compared to late-onset AD [54^▪▪^]. In AD, lower LC integrity correlated with greater endorsement of neuropsychiatric symptoms. Both the LC and the RN display lower metabolism, as measured by FDG-PET, in AD patients compared to controls [46,55].

Paradoxically, cerebrospinal fluid (CSF) NE levels are elevated in MCI and AD patients compared to controls, and these higher NE levels correlate with lower LC integrity, lower CSF Aβ and higher ptau [54^▪▪^]. These findings parallel previous CSF studies reporting elevated NE metabolites, such as MHPG, in cognitively impaired individuals [56,57], and suggest that LC neurodegeneration results in lower LC activity, but that synaptic changes, functional alterations to receptors or transporters in target regions, increase the availability of cortical NE. It can be speculated that the combination of elevated cortical NE, Aβ and tau may jointly spur neuronal hyperexcitability.

In AD models with cognitive impairment, LC neurons, RN neurons and their projections to the CA1, exhibit loss in neuronal excitability and spontaneous firing rates [58^▪▪^,59^▪^]. However, despite lower 5-HT levels in the RN [59^▪^], NE and 5-HT-metabolites were increased in the prefrontal cortex and hippocampus [58^▪▪^]. Chemogenetic activation of RN neurons increased inhibition of hippocampal excitability and reversed impairment of synaptic transmission and long-term potentiation, improved depressive-like behaviors and attenuated cognitive impairments [59^▪^]. Similarly, DREADD-induced LC activation restored learning in 16-month TgF344 rats [28] and optogenetic stimulation reversed memory deficits, monoaminergic neurotransmitters in the hippocampus and increased synaptic plasticity in 12-month-old 3xTgAD mice [60^▪^]. Treatment with bupropion, a NE and dopamine reuptake inhibitor in 5xFAD mice, showed improvements in LTP, memory, and reductions in Aβ [61^▪^]. These studies demonstrate that restoring NE or 5-HT reduces the synaptic toxicity of Aβ and improves memory impairment.

Thus, by the symptomatic stages of AD, substantial neurodegeneration of monoaminergic nuclei is evident, yet cortical NE levels remain elevated in the early symptomatic stages. This paradoxically complex dynamic likely reflects the interplay between failing local neuronal populations and cortical synaptic changes, which are likely detrimental and contribute to the hyperexcitability underlying Aβ-related tau spreading.

## TOWARDS A MONOAMINERGIC FRAMEWORK TO GUIDE RESEARCH AND INTERVENTION APPROACHES IN ALZHEIMER'S DISEASE

The monoaminergic systems represent a critical yet underappreciated frontier in understanding AD pathogenesis and developing intervention strategies. Converging evidence from neuropathological, animal, and human neuroimaging studies demonstrates that the LC and RN are not merely bystanders in AD progression but rather early and active participants whose dysfunction may catalyze downstream cortical pathology and clinical manifestations.

The temporal sequence emerging from this evidence is compelling: tau accumulation in these nuclei precedes cortical pathology by decades, triggering a cascade of morphological, functional, and neurochemical alterations that disrupt the delicate balance of monoaminergic signaling to cortical targets. These early changes, including altered excitability, neuroinflammation, reductions in dendritic density, and dysregulated neurotransmitter dynamics, occur well before neuronal loss and appear to drive both pathological spread and the emergence of neuropsychiatric symptoms.

The functional state of the neurons of these nuclei alters dynamically across disease stages, with evidence suggesting transitions from hyperactivity to hypoactivity [62]. Hyperactivity has often been interpreted as compensatory based on correlations with behavior. However, the evidence reviewed here indicates that sustained hyperactivity is excitotoxic, and while potentially beneficial for some behaviors (e.g. preserved cognitive function), it can be detrimental for others (e.g. neuropsychiatric symptoms). These seemingly opposing relationships may reflect parallel compensatory and detrimental effects, but could also be a result of methodological limitations, such as sampling biases, measurements not sensitive enough to detect subtle changes, or incomplete temporal windows. Furthermore, while many of these associations are described at the group level, they do not account for the substantial individual variability attributable to factors such as genetics, sex, or lifestyle variables. The inconsistencies in human neuroimaging findings underscore the complexity of measuring these small nuclei in vivo and highlight the need for more refined methodological approaches and longitudinal multimodal studies that can capture the dynamic changes and interactions across the disease trajectory.

**FIGURE 2:**
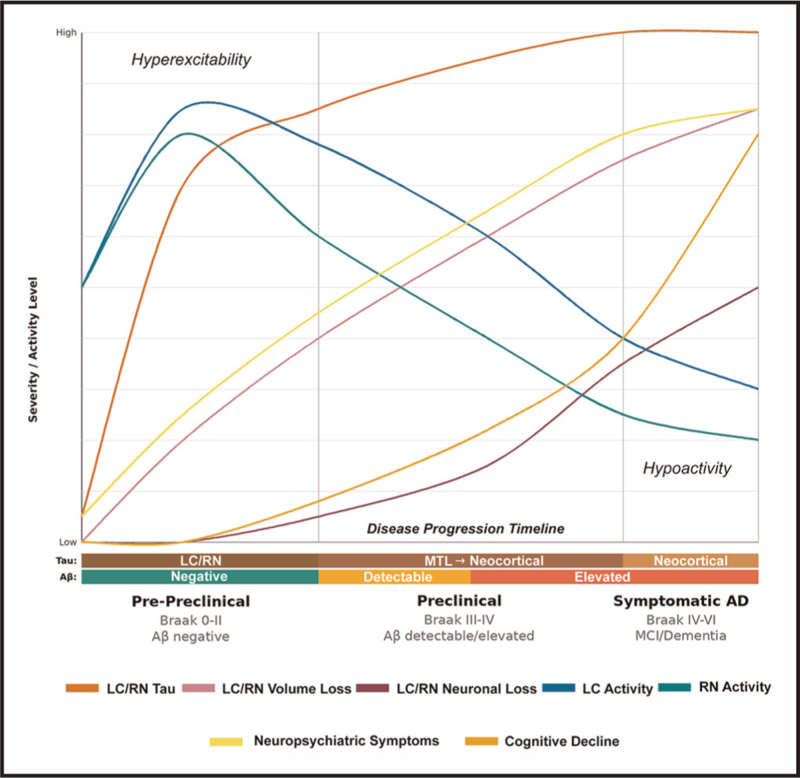
Trajectory of monoaminergic system alterations across Alzheimer's disease progression. Schematic outline of the framework linking morphological, functional, and molecular changes in the LC and RN to neuropsychiatric and cognitive changes during the disease stages of AD. Although the curves are based on the reviewed data, they are hypothetical and designed to guide research into modeling the temporal dynamics of these processes and their interactions. Tau accumulates early in adulthood in the LC/RN. This is associated with morphological changes (reductions in dendritic/fiber density, glial processes), but neuronal loss is observed in the preclinical stages (starting from Braak IV). Early in the asymptomatic stages, prior to the emergence of Aβ, LC and RN neurons showed increased firing rates (hyperexcitable), but as amyloid rises, these neurons become hypoactive. Cortical levels of NE and 5-HT are not depicted in this graph, as there is currently only data for the symptomatic stages, suggesting increases in NE and NE-metabolites and mixed evidence for 5-HT and its metabolites. These changes in the monoaminergic systems lead to neuropsychiatric symptoms and sleep-wake disruptions in the early stages of the disease, followed by cognitive decline. Together, this framework can provide novel insights into treatment strategies (specific vs. broad-spectrum, blocking or stimulating monoamines, combination therapies) based on neurotransmitter dynamics, disease stage, and symptom profile. LC, locus coeruleus; NE, noradrenergic; RN, raphe nuclei.

Based on the reviewed evidence, a framework summarizing the potential relationships between LC and RN changes and symptoms of AD across the disease trajectory is proposed in Fig. 2. In the earliest stages (prepreclinical), neurons in both nuclei accumulate tau, undergo morphological changes, and exhibit hyperexcitability. These pathologic changes contribute to the emergence of neuropsychiatric symptoms, such as depression, anxiety, sleep-wake disruption and initial tau progression to the earliest cortical regions in midlife. As Aβ rises and tau spreads through the medial temporal lobe regions to the first neocortical regions, marking the preclinical phase of AD, the RN and LC neurons become hypoactive and succumb; yet, cortical levels of neurotransmitters and their metabolites can remain elevated well into the cognitively impaired stages. Elevated cortical NE may increase tau phosphorylation and exacerbate hyperarousal-related neuropsychiatric symptoms, such as insomnia, fear or psychosis. The combination of elevated levels of cortical NE and Aβ further drives tau spreading and cognitive decline.

This framework holds important therapeutic implications. The monoaminergic system offers a range of FDA-approved medications that could be repurposed for AD. In a small phase II trial in AD patients, 6  months treatment with Atomoxetine showed mild improvement in cognition [63], in particular among those patients with higher scores, indicating that Atomoxetine may be more effective in earlier stages [63]. Recently, a 12-month, double-blind, placebo-controlled, crossover clinical trial in patients with MCI reported that Atomoxetine reduced CSF ptau [64] at rates similar to those of the FDA-approved monoclonal amyloid antibody, Lecanemab [65], suggesting opportunities for combination therapies.

Nonpharmacological interventions, such as transcutaneous vagus nerve stimulation, can be a safe, scalable option. Although the exact mechanisms of tVNS remain unclear, animal and neuroimaging data reported that tVNS increases LC and RN activity [66,67], possibly by co-stimulation of inhibitory pathways [68] or acting through serotonergic pathways to restore the excitation-inhibition balance. Vagus nerve stimulation in AD patients reduced CSF ptau levels and slowed cognitive decline, while improving memory in cognitively normal older individuals [69].

Crucially, given the dynamic changes in these neurotransmitters across disease stages, treatment selection must consider changes in these systems at different temporal and spatial scales, the disease stages, and the corresponding changes in symptoms. Dysregulation of neurotransmitters can have varying effects on behavior, where hypoarousal and hyperarousal can coexist, depending on the tonic-to-phasic ratio of neuronal firing and the activation/dysregulation of specific receptors and transporters. Therefore, precision therapeutics require more specific and targeted approaches instead of broad-spectrum treatments.

## CONCLUSION

As our field moves toward earlier intervention windows, the monoaminergic systems offer both a warning signal and a potential therapeutic target. The proposed monoaminergic framework can guide research studies examining temporal relationships between monoaminergic changes, AD pathology, and behavior at the individual level, and relate these observations to evaluate targeted intervention approaches. Understanding how to leverage these early vulnerability nodes may be essential for preventing or delaying Alzheimer's disease.

## Acknowledgements

*None.*

### Financial support and sponsorship

*This work was supported by the Alzheimer's Association (AARG-22-920434, PI: Heidi Jacobs) and NIH grants R01AG062559, R01AG06806, R01AG082006, and R21AG074220 (PI: Heidi Jacobs). This manuscript is the result of funding in whole or in part by the National Institutes of Health (NIH). It is subject to the NIH Public Access Policy. Through acceptance of this federal funding, NIH has been given a right to make this manuscript publicly available in PubMed Central upon the Official Date of Publication, as defined by NIH.*

### Conflicts of interest

*Heidi Jacobs is immediate past chair of the Neuromodulatory Subcortical Systems Professional Interest Area of ISTAART and served as advisory board member of ISTAART. These relationships are not related to the content in the manuscript. All other authors report no relevant conflicts.*

## REFERENCES AND RECOMMENDED READING

Papers of particular interest, published within the annual period of review, have been highlighted as:

▪ of special interest
▪▪ of outstanding interest
